# Mathematical Modeling and Validation of Retransmission-Based Mutant MQTT for Improving Quality of Service in Developing Smart Cities

**DOI:** 10.3390/s22249751

**Published:** 2022-12-12

**Authors:** Jawad Ali, Mohammad Haseeb Zafar, Chaminda Hewage, Raheel Hassan, Rameez Asif

**Affiliations:** 1 Department of Electrical Engineering, University of Engineering and Technology, Peshawar 25000, Pakistan; 2 Cybersecurity and Information Networks Centre (CINC), Cardiff School of Technologies, Cardiff Metropolitan University, Cardiff CF5 2YB, UK; 3 School of Computing Sciences, University of East Anglia, Norwich NR4 7TJ, UK

**Keywords:** QoS, MQTT, IoT, end-to-end service assurance, smart cities

## Abstract

Unreliable networks often use excess bandwidth for data integration in smart cities. For this purpose, Messaging Queuing Telemetry Transport (MQTT) with a certain quality of service (QoS) is employed. Data integrity and data security are frequently compromised for reducing bandwidth usage while designing integrated applications. Thus, for a reliable and secure integrated Internet of Everything (IoE) service, a range of network parameters are conditioned to achieve the required quality of a deliverable service. In this work, a QoS-0-based MQTT is developed in such a manner that the transparent MQTT protocol uses Transmission Control Protocol (TCP)-based connectivity with various rules for the retransmission of contents if the requests are not entertained for a fixed duration. The work explores the ways to improve the overall content delivery probability. The parameters are examined over a transparent gateway-based TCP network after developing a mathematical model for the proposed retransmission-based mutant QoS-0. The probability model is then verified by an actual physical network where the repeated content delivery is explored at VM-based MQTT, local network-based broker and a remote server. The results show that the repeated transmission of contents from the sender improves the content delivery probability over the unreliable MQTT-based Internet of Things (IoT) for developing smart cities’ applications.

## 1. Introduction

Physically separated and logically connected applications on distributed nodes are mostly used while developing smart cities, employing the IoE [1]. Such an application may include but is not limited to inventory and stock management, health and security, the entertainment industry, information segregation, information exchange, information integrity, autonomous functionality, transportation and traffic system, energy and smart gird, industrial manufacturing and process engineering, supply chain management, etc. The mode of communication can be Deice to Device (D2D) and Device to Server (D2S). D2D is based on the mechanism where devices are directly exchanging information for a certain process or service. On the other hand, in D2S or Server to Device (S2D), the distributed nodes are communicated with a server and the information exchange is on-demand. Integrated services often use a backbone server for an information exchange for various reasons.

For acquiring the optimum functionality of smart cities, a server must possess the required information or instructions that are timely needed by the connected devices [2]. Thus, for the stated purpose, abridged devices use various protocols, varying from application to application. The majority of these protocols serve as the foundation of the Internet of Things (IoT) and Internet of Everything (IoE). The Constrained Application Protocol (CoAP) is used for Machine to Machine (M2M) communication in smart energy and building automation. The Messaging Queuing and Telemetry Protocol (MQTT) [1] is used in manufacturing industries due to its open-source moldable nature, petroleum and natural gas, health, weather, home automation, smart farming, smart metering, remote, etc. Web Socket Protocol has a variety of uses such as in social media feeds, multiplayer games, collaborative editing/coding, clickstream data, financial tickets, media chats, location-based apps, online education, etc. [3]. The Data Distribution Service (DDS) is used on the top of edge devices/sensors for supervision, integration, and process control. Similarly, the Extensible Messaging and Presenting Protocol (XMPP) is widely employed in content syndication, collaboration tools, geolocation, file sharing, gaming, remote systems control, cloud computing, etc. These application protocols are the extensively used available protocols in the development of smart cities [3].

While talking about MQTT-based connectivity, it is worth noting that MQTT is used on the top of IEEE 802.15.4 and TCP/IP [4]. MQTT requires less power compared to other employed protocols for a range of applications where the power consumption accounts for the network performance. MQTT uses a traditional subscription and publishing-based mechanism, hence the bandwidth efficiency is managed up to a certain level.

In this work, the mathematical models for estimating the end-to-end delay and probability of the content delivery for retransmission-based mutant MQTT with QoS-0 are developed and then examined for their validation by analyzing the reliability of end-to-end MQTT. The attempts of sending information on a fixed subscription are varied per simulation and the results are then checked on a physical network. The emulated network nodes are comprised of fundamental sensing and connectivity features. The end nodes consist of Windows clients on PCs and as a Java application on handheld devices. The requests that are generated in a simulation environment on Linux are thus checked by a physical device for the performance consistency and validity of the mathematical model. The MQTT server is handled on a Virtual Machine, running at the eclipse.org cloud. The end nodes are connected to the Internet via a wired network, whereas the switches use optical fiber connectivity with the Internet. The DNS server is used in this case as well.

### 1.1. Architecture

**Figure 1:**
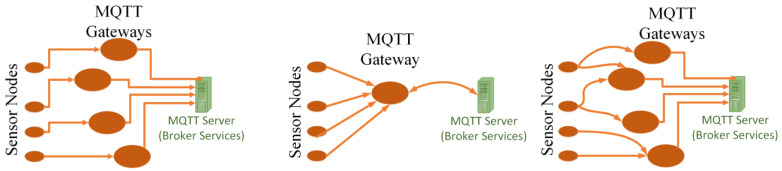
Logical Formation of MQTT gateways in IoT for various applications (**a**) Transparent Gateway MQTT, (**b**) Aggregated Gateway MQTT and (**c**) Hybrid Gateway MQTT.

MQTT is a subscription and publishing service-based communication with a centralized server [1]. The MQTT-based network is comprised of three basic logical formations, as shown in Figure 1. The transparent gateway has one node connection that is bridged with a remote MQTT server. The aggregating gateway connects local network nodes using a singular gateway to a remote MQTT server. Hybrid gateways route packets from multiple nodes and use mesh topology for connecting with the remote MQTT server [2]. The above-stated gateway formations are opted for various IoT scenarios. For example, a low-density network with large bandwidth requirements may employ the transparent gateway. This way, a single actuator–single sensor-based application may be run with a speedy response [1].

Each stated formation has its own facilities, uses and drawbacks. Aggregated gateways are not employed for the applications of smart cities when the nodes are geographically dispersed. It is because several nodes might be acting as actuators and controlling the nodes from physically separated locations, and this means that different MQTT gateways are used that makes the IoT network’s formation transparent (see. Figure 1a). Aggregated gateways have the limitations of poor congestion control and packet loss as well as a single point failure issue [1], directly degrading the performance of the applications of smart cities. On the other hand, a hybrid gateway, as in Figure 1c, uses multiple paths for communication with the server that will lack the concept of modularity for communication with a remote server on the Internet in our case. A Virtual Machine, while generating data traffic for the experiment, can only provide transparent gateway-based communication. Thus, the work uses the IoT network formation based on the transparent gateway-based client to server connectivity. The rest of the rules and assumptions are listed below.

- The MQTT subscribe/publish-based communication requests to the server are collectively treated as request–acknowledge messages.
- The end node uses DNS for address resolution in case of connectivity with the remote server that poses a constant delay.
- The SUBSCRIBER nodes as well as the PUBLISHER nodes follow an identical mechanism for communication with the server, thus the request flows through the same pathway.
- The TCP-based communication “fire and forget after *n* attempts” strategy is considered for all the network nodes that follows retransmission-based QoS-0.
- The remote server holds two sets of request queues. The published contents’ queue holds the data for the Tcnt interval and the subscribers’ requests are held for an interval denoted by Treq. These holding intervals Thold are thus defined by the storage capacity of the MQTT server as well as the connected nodes [2].
- QoS-1, that is based on “at least one time delivery”, and QoS-2, based on “exactly one time delivery”, are covered in [3].

### 1.2. Problem Statement and System Model

The services that are provided in smart cities are categorized by latency in the information exchange, bandwidth and packet loss. For example, the data communication networks that are used in a hospital environment must be in accordance with the severity of the patient’s condition [4,5]. Moreover, it may have several levels and priorities of information exchange w.r.t parameters that are being exchanged. For example, a single patient may require multiple services, each having different sets of the data transmission rate and data update rate. Similarly, the end-to-end delay for services at a normal condition may be compromised. For example, a remote weather station may exchange weather-related information with minimum assurance for its delivery in normal weather [1]. The reliability parameters need an upgraded communication scheme when the situation is of an elevated priority. For example, adverse weather condition may need an immediate data sharing service for smart decision making [6].

The research aims to assure an adequate reliability in terms of the content delivery and guaranteed end-to-end delay in the development of smart cities’ applications. Such applications use data from multiple sources/sensors on a single topic or on a range of topics for proper functioning that varies from application to application. The PUBLISH requests from sensing nodes are considered unique while receiving data, irrespective of their physical or logical situation [6]. Thus, the events are considered to have a mutually exclusive w.r.t delivery time or bandwidth requirements. Likewise, the logging of data on the centralized server always put a constrain on the residing time of the data as well as the queue size. We may term this as the packet expiry time or packet replacement time on the server. A single topic data may be required by different subscribers for a range of uses. For example, data from traffic junctions may be used for the re-routing of vehicles or for controlling traffic lights.

**Figure 2:**
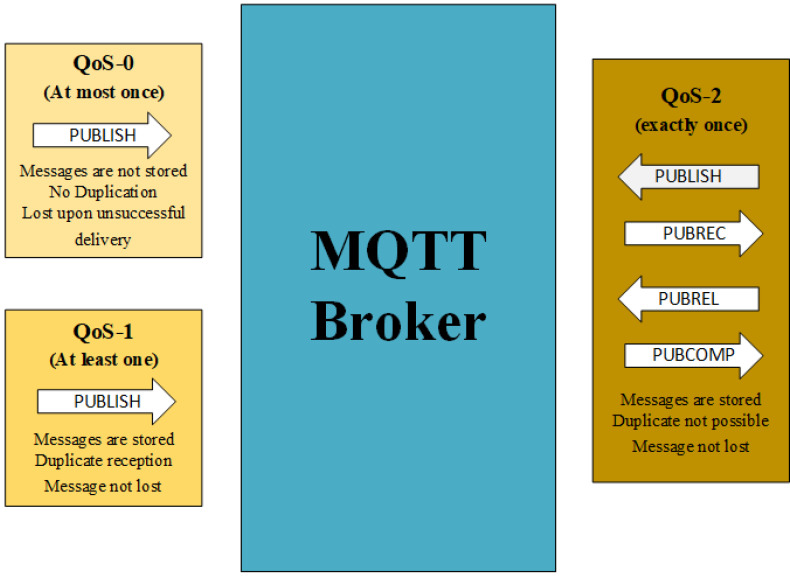
QoS-0-, QoS-1- and QoS-2-based MQTT broker with message delivery, duplication and storage status on the publishing node.

QoS-0, QoS-1 and QoS-2 are shown in Figure 2. In Figure 2, PUBLISH means post data to the MQTT Broker, and PUBREC means Publish has been Received, confirming the PUBLISH operation to a server in QoS-2 [3]. After receiving PUBREC, the sender sends a “Publish Release” request as PUBREL to tell the server that it has discarded the sent data from a local stack. The server also stored the packet identifier at this stage to avoid the processing of duplicate packets. The server responds with a confirmation packet of a Publish Complete (PUBCOMP) message after PUBREL. This way, the sender can repeat the process with new data. According to these QoS levels, either the packet is lost, or the packet is confirmed by an acknowledgement from the MQTT Broker server. The work proposes a one step ahead solution to improve the QoS of the MQTT-based smart cities’ connectivity applications. The MQTT protocol-based nodes are configured for a transparent gateway-based application. The nodes are running the simple process of publishing and subscribing services that are identical in nature. The work considers the number of connected nodes, the PUBLISH and SUBSCRIBE request rate, the time of content holding by the server and gateway for the retransmission-based QoS-0 MQTT IoT, for a system performance improvement in terms of the reliability and end-to-end delay. Here, a mathematical formulation is provided for achieving a guaranteed QoS by a varying number of retransmission attempts.

The rest of the work is organized as follows: Section 2 explains the basic literature and characteristics of MQTT. Likewise, the proposed retransmission-based MQTT is explained in the section. Section 3 is the mathematical modeling of the end-to-end delay and expressions for evaluating the probability of the content delivery. The experimental setup and system model is given in Section 4. Section 5 provides a complete insight into the obtained simulation results and its explanation w.r.t scalability of the network. Section 6 concludes the work and gives future perspectives on this research.

## 2. MQTT Characteristics

MQTT is light weight and content-oriented protocol. Locally, MQTT can be used for the IoE and IoT, i.e., amongst IP-less as well as IP-based network nodes. The explored case uses the wired network of IP-based nodes that are connected to a local gateway on a single node–single gateway scheme basis. Mosquito clients are deployed on local nodes in an emulated environment for real world connectivity by providing the NAT to LAN bridge with the Internet. This provided real world environment, sufficient to receive effective and authentic readings of the parameters that are under observation.

### 2.1. Retransmission-Based QoS-0 MQTT Mutant Protocol

By the mechanism of retransmission in the QoS-0, the instantaneous delivery probability, that is explored for the static network characteristics in [5], is affected. The requestee node retransmits the TCP/IP-based request if the packet is either lost between the node and gateway or on the Internet. Additionally, the gateway data buffer as well as the request queue at the server are kept of a minimum size. It is for the fact that the MQTT gateways usually have a limited cache for storing data. The queue is only handled at the server, so the gateway limitations are traded off by retransmit protocol. This process makes the midway delays static for usual processes and define (make constant) it instantaneously.

### 2.2. Proposed Asynchronous MQTT

**Figure 3:**
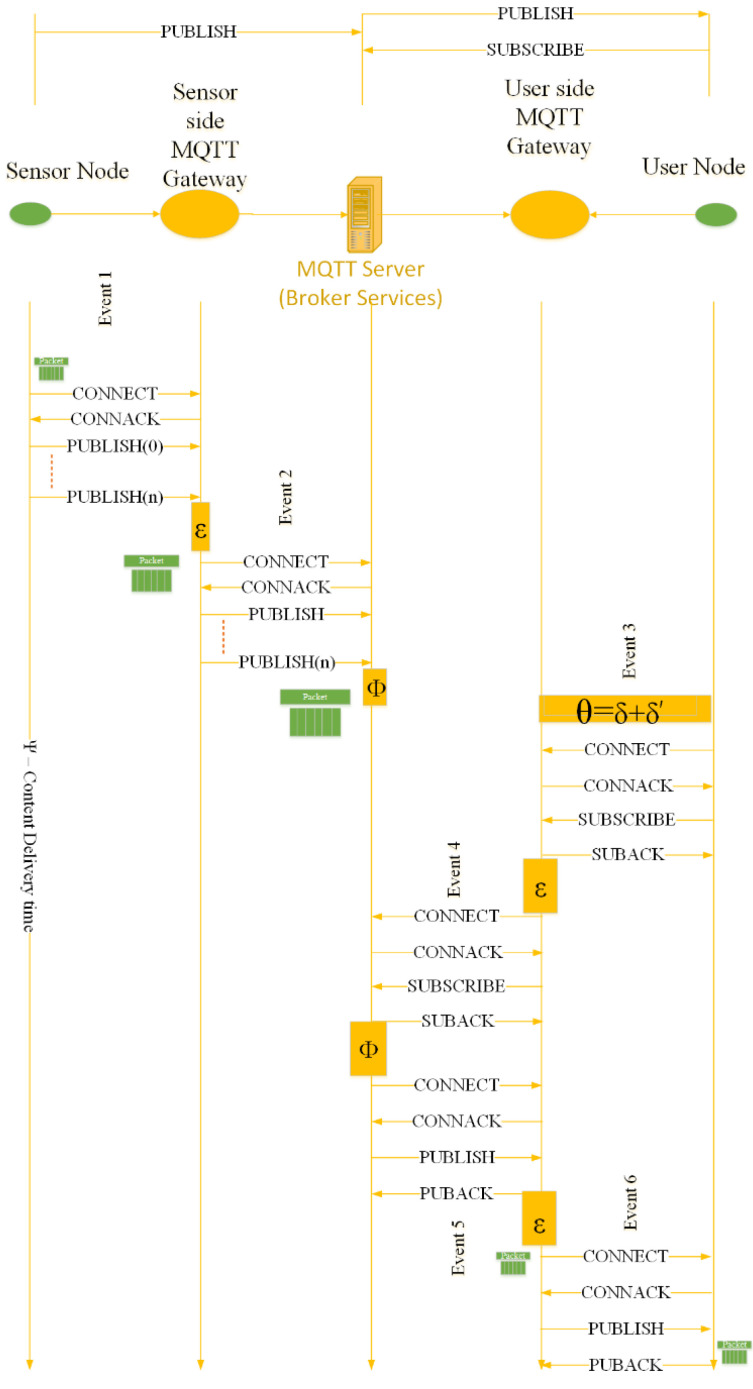
End-to-end delay and packet travel system model for QoS-0 retransmit Mutant MQTT.

We have assumed that one node is posting for a single topic and multiple topic-based posting is assumed to be separate nodes and separate events. The rest of the communication scheme between the publisher and subscriber node is explained in the following steps, as in Figure 3.

**Step 1:** The end node connects with the gateway using the CONNECT/CONNACK mechanism and then we publish the contents using the publish–retransmit mechanism.
**Step 2:** The received data from the end node is transmitted by the gateway to the remote MQTT server as CONNECT/CONNACK and then a publish–retransmit basis over a reliable TCP/IP wired network.
**Step 3:** The end user connects to the gateway using the CONNECT/CONNACK mechanism as in Step 1 and then the same end user registers with the gateway using SUBSCRIBE and SUBACK for an already available topic on the server.
**Step 4:** The subscriber side gateway registers the end user with the server using the connect request followed by the subscribe request that is acknowledged by the server as the SUBACK.
**Step 5:** The server sends the available date of the subscribed topic to the end user side gateway via the Internet using PUBLISH and PUBACK instructions.
**Step 6:** The gateway on the end user’s side fitches the data to the end user using PUBLISH and PUBACK.

The above-stated steps pose a certain probability of failure for a send and forget mechanism. This send–retransmit and forget mechanism is used for the asynchronous IoT system in Figure 3. The availability of the data on the server is arguable.

## 3. Delay Estimation

The probabilistic model uses an asynchronous mode of data transfer for the collection and distribution of data. Contents are created at sensors using a constant request rate that are sent to the intermediate gateway with a send–retransmission and forget manner. The same method is followed for fetching the sensors’ data to a remote MQTT server from the intermediate gateway. There are two cases for which an end-to-end delay may be estimated while considering the availability of the contents on the server for the requested topic from the end user.

The probability of one successful delivery of a data packet from the sender to receiver is comprised of the exclusive probabilities of two event probability, i.e., the sender to server probability and server to subscriber probability. Now that the subscriber does a handshake in TCP/IP-based data fetching, the number of total attempts are denoted by ‘*i*’ for relating towards the packet probability p_f_, and the number of acknowledgements from the communicating node is ‘*j*’ for relating the reverse packet probability *p_r_*, whereas the number of maximum iterations from the sender to server are considered to be ‘*n*’ for the sensor to the gateway and ‘*m*’ for the gateway to the server.

We have considered that attempts m and *n* are equal. The success probability is given by (1) as:

*P*(*i,j*)= *p_f_^j^.* (*1* − *p_f_*)*. p_r_^i^.* (*1* − *p_r_*) (1)

### 3.1. Contents Available on Topic

The iteration-based packet sending probability for *n* events of retransmission is given by (2):

*P*(*n*) = *∑P_f_*(*i ≤ n*) (2)

A single transmission/retransmission is completed after the delay time *td*. This ‘*td*’ must be greater than the product of the request rate ‘*σ_k_*’, where *k* is a unique node and the time taken to completely send one request *T_r_*. This shows that the total requests after the retransmission will take time, as in (3).

*Tr*(*n*) = *nσktr* (3)

The point-to-point latency also includes the synchronization time *T_s_*, that is a single packet traversing time between the two nodes and the server on the end user’s side using TCP. The latency of a successful packet delivery is given by (4)–(6).

(4) $$Lt\left(i,j\right)=Lt\left(n\right)=unsuccessfulattempts+successfulattempts$$

(5) $$L_{t}\left(n\right)=\left(n\right)\sigma_{k}\cdot t_{r}$$

(6) $$L_{t}\left(i,j\right)=RTT+\left(\overset{i-1}{\underset{k=1}{\sum }}2^{k}T_{s}\right)+\left(\overset{j-1}{\underset{l=1}{\sum }}2^{l}T_{s}\right)$$

The overall probability of latency *P*[*L_t_*(*n, i, j*) *≤t*] on a low data rate channel for the duration *t* is:

(7) $$P\left[L_{t}\left(n,i,j\right)\leq t\right]=\underset{Lt\left(i,j\right)}{\sum }P\left(i,j\right)+\underset{Lt\left(n\right)}{\sum }P\left(n\right)$$

For the fact that two additional TCP connections are established, we assume a *ε* queueing delay at the gateways. The gateway may hold sensing data to be forwarded to the MQTT server or it may enqueue the date while sending it to the end node. The delay posed by the MQTT server is denoted by *Ф*. If the data are already available on the server, two cases may arise. A sensing node will either send a publish request or an end node will make a request for the contents. The connection request delay for a subscription service is denoted by *δ*.

The content delivery time *Ψ* for a successful route traversal from the sensing node *n* via the sensing side gateway *g*, through the server s and then from the server to the end user *u* through another gateway *g*, is given by (8)

*Ψ* = *T_connect-ng_* + *T_pub-ng_* + *ε* + *T_connect-gs_* + *T_pub-gs_* + *Ф* + *δ* + *T_connect-ug_* + *T_sub-ug_* + *ε* + *T_connect-gs_* + *T_sub-gs_* + *Ф* + *T_connect-sg_* + *T_pub-sg_* + *ε* + *T_connect-gu_* + *T_pub-gu_* (8)

By examining (7) w.r.t Figure 3, we may replace the *T_connect-p2p_* by *RTT_connect_*, *T_pub-ng_* and *T_pub-gs_* with $2\left(n\right)\sigma_{k}t_{r}$, *T_pub-sg_* and *T_pub-gu_* with *RTT_pub_* and *T_sub-p2p_* with *RTT_sub_*. Thus, Equation (8) is express as:

(9) $$\Psi =6{\mathrm{RTT}}_{\mathrm{connect}}+2\left(n\right)\sigma_{k}t_{r}+2{\mathrm{RTT}}_{\mathrm{pub}}+2{\mathrm{RTT}}_{\mathrm{sub}}+3\varepsilon +2Ф+\delta$$

Since PUBLISH requests are always of the same length. Moreover, the connection requests are identical. So, by keeping a fixed packet size for all the TCP requests, we may combine *RTT_connect_*, *RTT_pub_* and *RTT_sub_* and thus receive (10) for a collectively called *RTT*, the TCP Round Trip Time for each event:

(10) $$\Psi =10\mathrm{RTT}+2\left(n\right)\sigma_{k}t_{r}+3\varepsilon +2Ф+\delta$$

### 3.2. Contents Not Available on Topic

The constant *δ* in (10) represents the queueing delay experience by the subscription request faced at the server before the content is sent to the end user. If the contents on a requested topic are not available, the subscription request may be answered but the user will have to wait for a *δ′* duration before the request is answered by the server. We may collectively call this queueing delay *θ*, and it is calculated by adding *δ′* with *δ*, as in (11).

(11) $$\Psi =10\mathrm{RTT}+2\left(n\right)\sigma_{k}t_{r}+3\varepsilon +2Ф+\theta$$

### 3.3. Expressions for θ, ε, RTT

*Θ* may be given by a uniform distribution between the two events, i.e., the arrival instant of content *Ť_cnt_* and the arrival time of request *Ť_req_*, at the server. This interval is given by (12)

*θ* = [*Ť_cnt_,Ť_req_*] (12)

We will use the relation from [6,7,8], meaning the random delay from the gateway to the gateway to the server is given by (13):

*μ_θ_* = (*t_cnt_* + *2T_cnt_* − *δ*)*/2*] (13)

Also, for the contents already available on the server:

*θ* = *δ* = *3 RTT* + *ε* (14)

Also, the Smooth Round Trip Time is given by (15), thus the *RTT* is calculated as (16). In (15) and (16), 0 < *β* < 1 and 0 < Δ < 1 and uses *β* = 0.125 for a smooth *RTT*, as suggested by [6,7,8].

*SRTT*[*i*] = (*1* − *β*)*x SRTT*[*i* − *1*] + *βxRTT* (15)

Additionally,

*RTT* = *RTT* + *Δx Diff,…, Diff* = *SRTT* − *RTT* (16)

### 3.4. Service Rate, Capacity of the Network, and Probability of Content Delivery

The queueing delays can be calculated from the virtual service rate *S_rate_*. For connected users *N_c_*, the service rate’s threshold is given by relation in (17):

(17) $$\overset{Nc}{\underset{i=1}{\sum }}\lambda_{i}\leq S_{rate}$$

(17) gives us the average processing time for the contents in terms of the arrival rate *λ_i_*. For the packet length *L*, the service duration *s_i_* is calculated in (18).

*s_i_* = *L/μ_i_* (18)

here,

(19) $$\mu_{i}=\frac{\lambda_{i}}{\sum_{i=1}^{Nc}\lambda_{i}}S_{rate}$$

The request arrival rate is given by (20):

(20) $$\mu_{i}{}^{req}=\frac{\sigma_{i}}{\sum_{i}^{Nr}\sigma_{i}}S_{rate}$$

The maximum queueing delay by both the content and request is given by *Q*[*λ_i_, σ_i_*] in (21), as:

(21) $$Q\left[\lambda_{i},\sigma_{i}\right]=\frac{2-\rho_{i}{}^{cnt}}{2\mu_{i}\left(1-\rho_{i}{}^{cnt}\right)}+\frac{\left(2-\rho_{i}{}^{req}\right)\rho_{i}{}^{req}}{2\left(1-\rho_{i}{}^{req}\right)}$$

Here, $\rho_{i}{}^{req}=\sigma_{i}/\mu_{i}$ and $\rho_{i}{}^{con}=\lambda_{i}/\mu_{i}$.

The probability of the content delivery for the M/D/1 fixed scheduler-based queue model, having *D* = *1/μ*, is given by (22), as per [9,10].

(22) $$P_{cnt}^{D}=\frac{\sigma_{i}}{2}\left(\left[1-e^{-\zeta_{i}/Tcnt}\right]+\left[1-e^{-\zeta_{i}/Treq}\right]\right)$$

## 4. Experimental Setup

**Figure 4:**
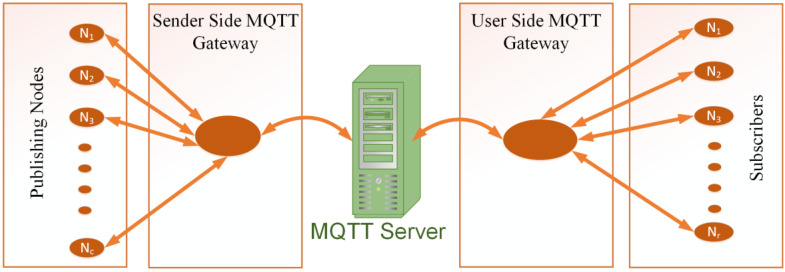
System Model and connectivity.

The test bed is comprised of an emulated environment for remote nodes that are connected to the MQTT broker, as in [11]. Linux-based nodes are used for creating topic-based data at various data rates. Mosquitto MQTT clients are deployed on Virtual Machines for this purpose. The VMs consist of a range of clients and subscribers to approximate the scenario for the practical results and a realistic environment. The Mosquitto MQTT broker is used on a local server and an Eclipse IoT server. The clients from windows, android and VMs are subscribed to various topics on this server. The setup of the nodes is given in Figure 4.

The paper assumes that Body Area Network (BAN) nodes are connected nodes. The data of various patients are collected and then the subscribers’ requests for the collected data, as in [12]. This work is not limited to a single patient or specific parameter because MQTT supports topic-wise data and different patients can be dealt as different topics on the server. For the purpose of the analysis, we have used a temperature sensor, heartbeat sensor, moisture sensor and fall detection sensor data as the primary source.

The arrival rate of contents *λ* by the sensing nodes and requests rate for the contents *σ* by the subscribers are treated as the same. Both rates are varied between 100 kbps and 2 Mbps, with no retransmission involved. The client node traffic uses a Constant Bit rate (CBR), which is varied using the Poisson distribution mean [13]. The *RTT* for TCP is obtained by the terminal as the ping6 command, whereas for windows, the power shell is used. These values are saved to a comma separated value (csv) file for a further analysis. The gateway queueing delay is kept at 720 µs, as in [14]. The payload is set indirectly by CBR [15]. The local server keeps the request for the contents and the contents on the server for 10 ms as a fair queueing policy [14]. The M/D/1 queue is used at the local server. Ref. [11] has used an infinite queue size but due to hardware-based servers, we are limiting the dedicated local server capacity to 5 GB and the Mosquitto server on Eclipse.org uses an 80 GB shared capacity-based service.

## 5. Results and Discussion

**Figure 5:**
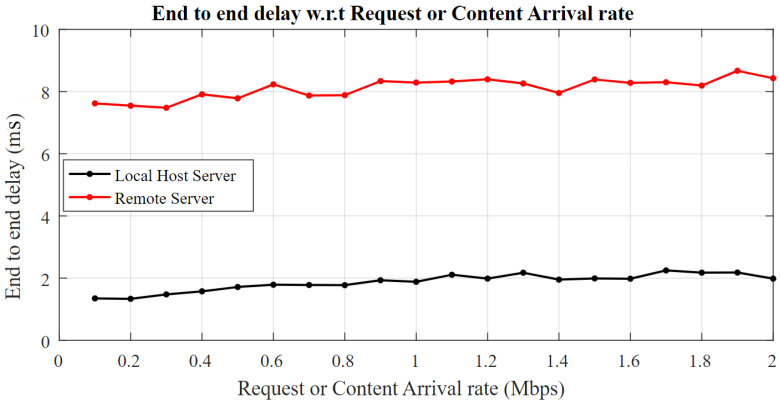
End-to-end delay for request and content arrival.

Figure 5 is a graphical depiction of the conclusion drawn by (10). The request rate or content arrival rates are controlled by the connected nodes in the IoT. Starting from 100 kbps, these rates are incremented with a step of 0.1 Mbps up to 2 Mbps. Moreover, the request arrival rate *σ* and content arrival rates *λ* are kept the same for the fact that the behavior of both the content provider and subscriber nodes is the same. The local server gives an end-to-end delay of 1.6 ms for an arrival rate of 100 kbps. The behavior remains linear, and the end-to-end delay increases to 2 ms for an arrival rate of 2 Mbps. The results are satisfactory as compared to the work in [11]. The remote server poses a 7.7 ms delay for 100 kbps traffic, whereas the delay slightly increases to 8.5 ms for a 2 Mbps request rate. Nonlinearity is seen between the 0.4 and 0.5 Mbps and the 1.0 and 1.4 Mbps request arrival rate in Figure 5. It is for that fact that VMs share their network and processing power for other processes. Similarly, the remote server is accessed through the Internet over a shared broadband network that causes the unavoidable nonlinearity in the graphs.

**Figure 6:**
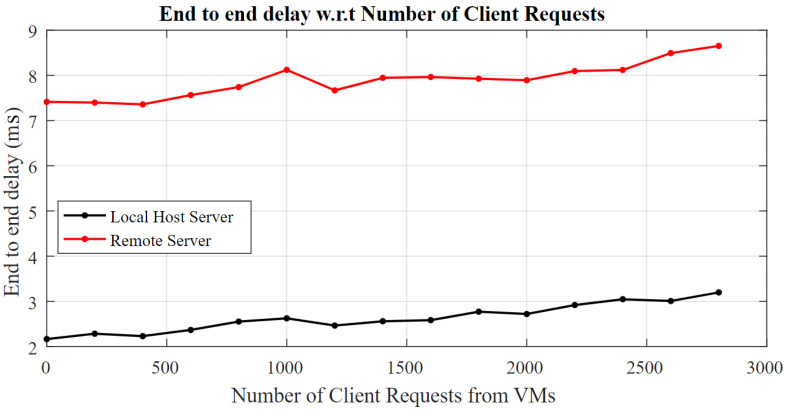
End-to-end delay w.r.t number of client request from connected IoT nodes.

The end-to-end delay w.r.t number of topic subscriptions or the clients’ requests is also explored for the real time traffic that is graphed in Figure 6. The delay in the delivery is linearly increasing while varying the number of client requests between 1 and 2800. The arrival rate is kept at 1 Mbps. The end-to-end delay increments from 2.2 ms to 3.2 ms, likewise for a locally deployed server. The delay steps up to 7.4 ms when remotely accessing the server, for which the maximum delay of 8.65 ms is experienced when 2800 requests are handled by the remote server. This also concludes that one request is handled at 500 μs on average on the server and a total service time (Δt_d_) of 1.4 ms is utilized for all the requests. The nonlinearities that are caused by shared processes on the VM and broadband network can be seen between the number of nodes ranging from 1000 to 1200.

**Figure 7:**
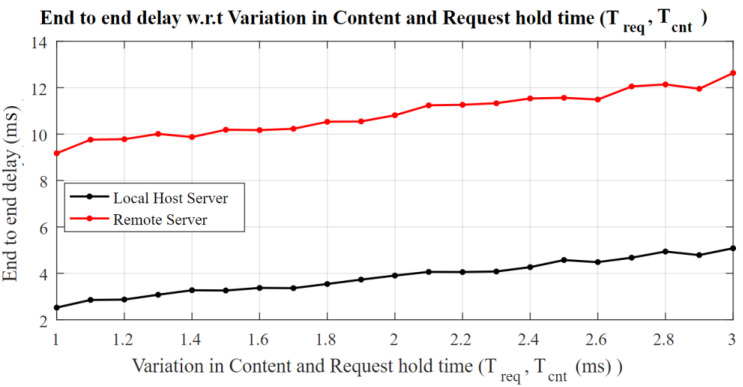
End-to-end delay w.r.t variation in content request time and content holding time.

Figure 7 explores the content holding time and gives a notion about the end-to-end delay w.r.t *T_cnt_*, and the delay increases drastically with the request hold time on the server. An average of a 300μs delay is introduced by increasing the hold time by 50 ms. The slope of the line increases with an increase in the round-trip path through the network.

**Figure 8:**
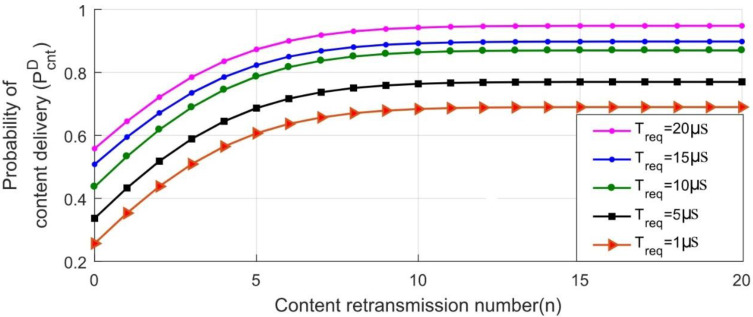
Probability of content delivery on localhost MQTT server over Virtual Machine for content interval (*T_cnt_*).

**Figure 9:**
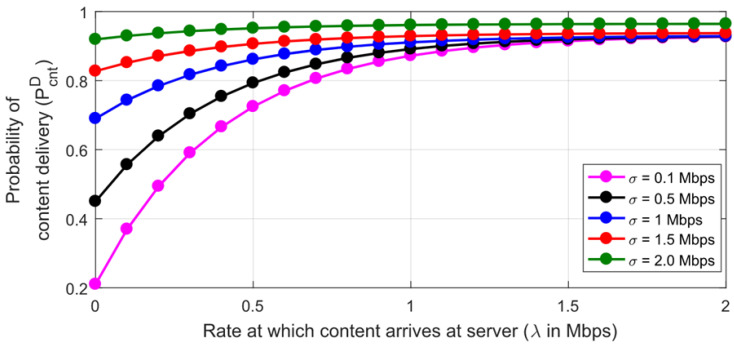
Effect of content arrival rate on probability of content delivery for *n* = 1.

The analysis probability of the content delivery *P_D_^cnt^* is analyzed; w.r.t content retransmissions *n* and *σ* is given by Figure 8 and Figure 9. Both figures can be directly used as a benchmark for the scalability of smart cities’ services. The request duration is incremented from 1 µs to 20 µs with a 4–5 µs step, likewise. It is worth noting that *P_D_^cnt^* linearly improves by increasing the *n* or *Treq* on the server. Additionally, the maximum achievable *P_D_^cnt^* can be directly calculated for the various set of retransmission attempts *n*. While keeping *Tcnt* constant, increasing the arrival rate of the contents also improves the delivery probability, as depicted by Figure 9. The results show an improvement in the achieved maximum probability of the delivery for a 2 Mbps arrival rate in [11] by 4%. The results show that the behavior of the developed scheme exponentially converges the performance of the scalable IoT.

**Figure 10:**
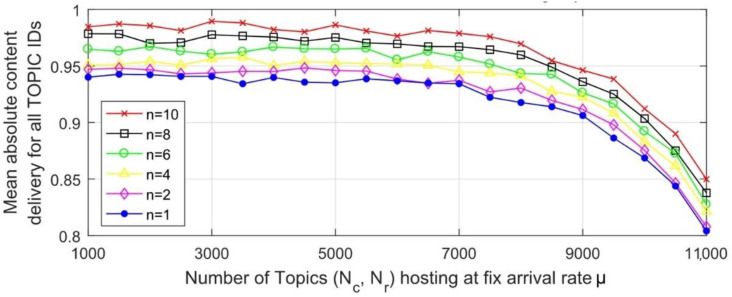
Mean absolute content delivery for collective topic IDs w.r.t connected hosts at fixed arrival rate for *n* = 1, 2, 4, 6, 8, 10.

Figure 10 is a broad picture of the developed scheme where the number of connected nodes is varied between 1000 and 11,000. The figure is a depiction of the real time content delivery and service degradation matrix. The remote services are degraded by 13%, whereas a 4% degradation is noted for the locally hosted MQTT server.

The portrayed image of the mean absolute content delivery for all the topic IDs is thus conformation that the degradation is practically tolerable for the massively deployed–re-motely accessed server with a retransmission-based mutant MQTT scheme.

## 6. Conclusions and Future Perspectives

The paper has proposed a mutant QoS-0-based model that examines the retransmission of a guaranteed end-to-end service, the probability of the content delivery and the mean absolute content delivery w.r.t number of the connected nodes (*Nc* and *Nr*), arrival rates (*λ* and *σ*), content hold time (*Tcnt*) and content retransmission *n*. The parameters are first used in the modeling system performance equations and then examined in a real time environment. Figure 10 bears more importance of all because it directly gives us a clear picture about the scalability of the IoT. Thus, smart cities can use the concept directly from this graph. A lower Round trip time *RTT* can be achieved with a lesser means absolute content delivery while keeping the value of *n* at the minimum. Likewise, a guaranteed packet loss is only possible when certain *λ* and *σ* are used in this regard.

Table 1 gives a comparative analysis of the proposed model, i.e., QoS-0 retransmission (for *n* = 1 and *n* = 10) with the currently employed MQTT service models. The CPU usage and transmission latency clearly state that the developed mechanism of mutant QoS-0 retransmission MQTT outperforms both in terms of the scalability as well as the guaranteed QoS with the bare minimum usage of the extra CPU power. The developed QoS-0 retransmission MQTT gives an improved packet delivery with the minimum repetition of the publishing process. Although the JoramMQ is promising for a lossless network for utilizing 3% of CPU power, it is a single transmission and discard-based protocol that degrades the packet delivery drastically over a lossy network. The work is a modified version of the Mosquitto Server scheme and reduces the CPU usage by more than 50% for an “at most once” MQTT mechanism, as in Figure 2.

The future of this work may go in three directions. One can include the re-transmission and acknowledged scheme in QoS-1 services with a fixed rule for the retransmission and for checking the improvement in the availability of the contents at the remote server. The CPU usage, that is not dealt due to the availability of a continuous power source in this work, is also debatable. A lower content delivery using a UDP-based network can be improved by applying same mathematical model of calculating the end-to-end delay of the network; this way MQTT-SN can be merged with retransmission-based mutant MQTT to reduce the network overheads, as in [4].

## Figures and Tables

**Table 1:** Comparison of the improved MQTT protocol with Benchmark MQTT, JoramMQ [16], Apollo servers [16], and RabbitMQ [17].

Service	Parameter	Apollo	JoramMQ	RabbitMQ	Mosquitto Server	QoS-0 Retransmission MQTT (*n* = 1)	QoS-0 Retransmission MQTT (*n* = 10)
QoS-0	CPU Usage	6%	3%	38%	24%	11%	15%
	Message Transmission Latency	5 ms	1.5 ms	10 ms	10 ms	2 ms	3.4 ms

## Data Availability

Not applicable.
